# Fast-GPU-PCC: A GPU-Based Technique to Compute Pairwise Pearson’s Correlation Coefficients for Time Series Data—fMRI Study

**DOI:** 10.3390/ht7020011

**Published:** 2018-04-20

**Authors:** Taban Eslami, Fahad Saeed

**Affiliations:** Department of Computer Science, Western Michigan University, Kalamazoo, MI 49008, USA; taban.eslami@wmich.edu

**Keywords:** fMRI, Pearson’s correlation coefficient, GPU, CUDA, matrix multiplication

## Abstract

Functional magnetic resonance imaging (fMRI) is a non-invasive brain imaging technique, which has been regularly used for studying brain’s functional activities in the past few years. A very well-used measure for capturing functional associations in brain is Pearson’s correlation coefficient. Pearson’s correlation is widely used for constructing functional network and studying dynamic functional connectivity of the brain. These are useful measures for understanding the effects of brain disorders on connectivities among brain regions. The fMRI scanners produce huge number of voxels and using traditional central processing unit (CPU)-based techniques for computing pairwise correlations is very time consuming especially when large number of subjects are being studied. In this paper, we propose a graphics processing unit (GPU)-based algorithm called *Fast-GPU-PCC* for computing pairwise Pearson’s correlation coefficient. Based on the symmetric property of Pearson’s correlation, this approach returns N(N−1)/2 correlation coefficients located at strictly upper triangle part of the correlation matrix. Storing correlations in a one-dimensional array with the order as proposed in this paper is useful for further usage. Our experiments on real and synthetic fMRI data for different number of voxels and varying length of time series show that the proposed approach outperformed state of the art GPU-based techniques as well as the sequential CPU-based versions. We show that Fast-GPU-PCC runs 62 times faster than CPU-based version and about 2 to 3 times faster than two other state of the art GPU-based methods.

## 1. Introduction

Functional magnetic resonance imaging (fMRI) is a non-invasive brain imaging technique which is used by researchers in order to study functional activities of the brain [1]. Using this technology, many facts about the brain are discovered based on blood oxygen level dependent (BOLD) contrast. Analyzing fMRI data using machine learning techniques for discovering hidden patterns and early-stage detection of several brain-related diseases has gained significant attention among fMRI researchers [2,3]. During an fMRI session, a sequence of images are taken by a scanner through time while subject performs one or more tasks (task-based fMRI) or the subject just rests without falling asleep (resting state fMRI). The fMRI data consists of several thousands or millions of very small cubic components called *voxels*. Each voxel is the smallest addressable element of the brain and houses millions of neurons inside it. Hemodynamic changes inside the brain are revealed as intensity changes of the brain voxels [4]. By keeping track of intensity of each voxel over time, a time series is extracted out of each voxel which is used for further analysis. A popular technique for analyzing brain functional connectivity is Pearson’s correlation coefficient (PCC) [5,6,7]. The PCC computes linear association between two variables *x* and *y* using the following formula:

(1) $$\rho_{xy}=\frac{\sum_{i=1}^{T}\left(x_{i}-\bar{x}\right)\left(y_{i}-\bar{y}\right)}{\sqrt{\sum_{i=1}^{T}{\left(x_{i}-\bar{x}\right)}^{2}}\sqrt{\sum_{i=1}^{T}{\left(y_{i}-\bar{y}\right)}^{2}}}$$

The value of PCC $\rho_{xy}$ can be in the range −1 and 1 [8]. Value of −1 indicates perfect negative linear relationship, 0 indicates no linear relationship and +1 shows perfect positive linear relationship among two variables. In this equation *x* and *y* correspond to two *T* dimensional variables. Considering fMRI data, *x* and *y* are two individual voxels each having *T* data points in their time series. Covariance and its normalized version correlation are useful measures for analyzing and studying brain functional associations. Jiao et al. [9] proposed multilayer correlation maximization (MCM) model for improving steady-state visual evoked potential (SSVEP) recognition accuracy in brain–computer interface application. Our quantitative experiments have shown that Eigenvalues and Eigenvectors computed from the covariance matrix of brain regions are discriminative metrics for classifying subjects with attention deficit hyperactivity disorder (ADHD) disorder from healthy subjects [10]. One of the most important applications of Pearson’s correlation is estimating functional connections and hence they are used to define edges among different regions or voxels of the brain [11]. Zhang et al. [12] used correlation for constructing their proposed framework hybrid high-order functional connectivity (FC) networks to diagnose early mild cognitive impairment disorder. Zhao et al. [13] used Pearson’s correlation for constructing the brain functional network and investigated its topological properties for Alzheimer diseased and healthy subjects. Godwin et al. [14] used this measure for defining connectivity between different regions for studying the functional connectivity during a working memory task in patients with schizophrenia. It is also used by Baggio et al. [15] for defining edges in functional network of subjects to study how Parkinson’s disease changes the global and local measures of the network. Another well-used application of Pearson’s correlation is proposed by Craddock et al. [16] for parcellating the whole brain fMRI data to spatially coherent regions such that voxels in each region have homogenous functional behavior. In this technique, Pearson’s correlation is used for computing functional connectivities among neighboring voxels and spectral clustering is applied to the resulting graph. Due to the compute- and data-intensive nature of calculating pairwise PCC among all voxels of the brain, brain researchers tend to seek more simplified models of the functional connectivity. Such models include considering groups of neighboring voxels or regions instead of each individual voxel or applying spatial constraints in their algorithms which can cause loosing useful information compared to voxel level analysis. The high time complexity is more hindering for studies involving a group of subjects since pairwise correlation should be computed for each subject separately. Time consuming nature of this process for large datasets leads us to use parallel computing techniques. Several parallel computing based approaches have been proposed in order to accelerate the PCC computation. One of these approaches is a graphics processing unit (GPU)-based approach proposed by Gembris et al. [17]. They reformulated the Pearson’s correlation equation in order to minimize the number of necessary divisions as follows:

(2) $$\rho_{xy}=\frac{T\sum_{i=1}^{T}x_{i}y_{i}-\sum_{i=1}^{T}x_{i}\cdot \sum_{i=1}^{T}y_{i}}{\sqrt{T\sum_{i=1}^{T}x_{i}^{2}-{\left(\sum_{i=1}^{T}x_{i}\right)}^{2}}\sqrt{T\sum_{i=1}^{T}y_{i}^{2}-{\left(\sum_{i=1}^{T}y_{i}\right)}^{2}}}$$

**Figure 1:**
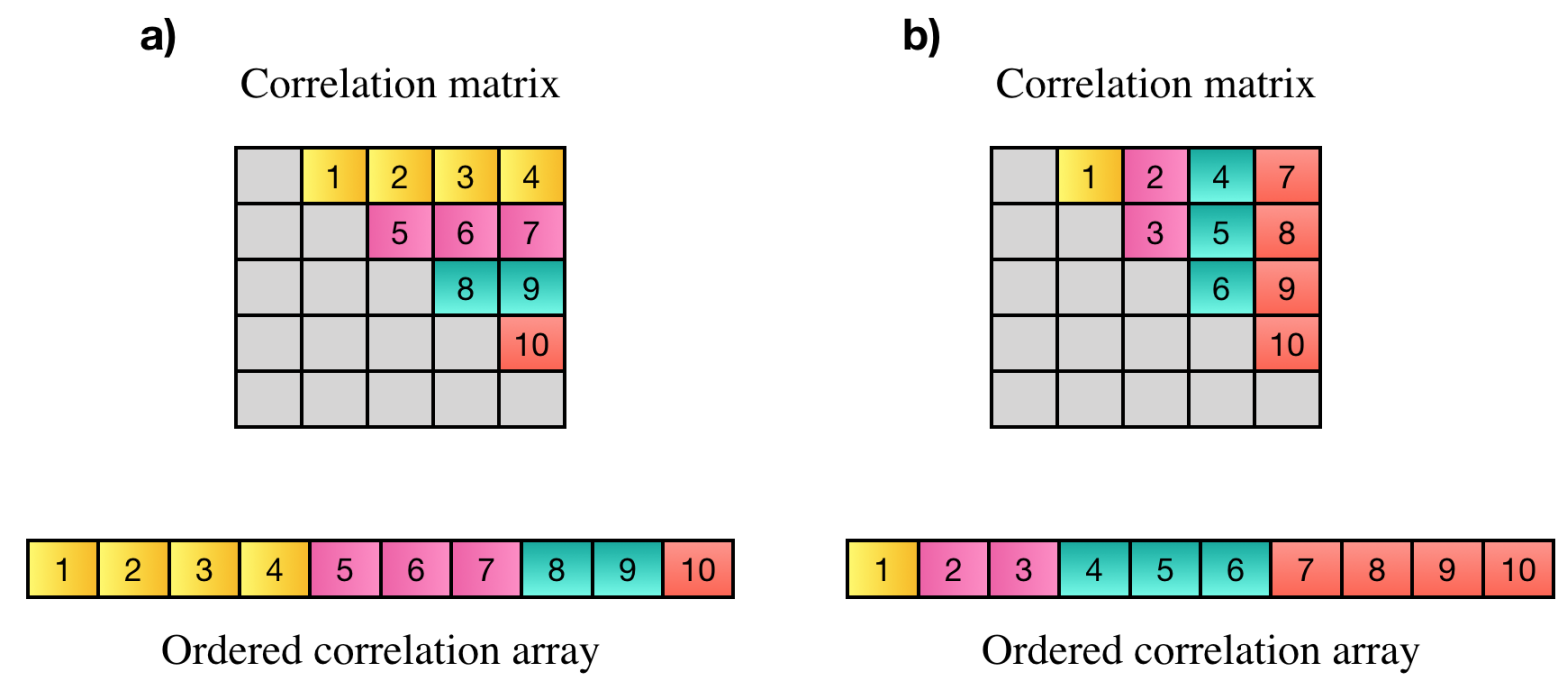
(**a**,**b**) Are examples of two possible orders for PCC matrix and their resulting correlation array. In part (**a**), the first N−1 elements of the array show the Pearson’s correlations between the first variable and all other variables, the next N−2 elements show the correlation of the second variable with all others and so on. In part (**b**), the last N−1 elements show correlation of the last element with the rest of elements, N−2 elements before them show the correlation of the N−1th element to the rest of elements and so on.

Wang et al. [6] proposed a parallel technique based on a controller worker method with message passing interface (MPI) to compute pairwise Pearson’s correlations over multiple time windows. Another approach was proposed by Liu et al. [18] to compute all pairwise correlation coefficients on Intel Xeon Phi clusters. Pearson’s correlation has symmetric property (corr(x,y)=corr(y,x)). Based on this property all pairwise correlations among *N* elements can be represented by an array of N(N−1)/2 elements instead of $N^{2}$ elements. Each element of this array is the correlation among two distinct variables *i* and *j*. The correlation array may contain all correlations in strictly upper or lower triangle part of the correlation matrix. Elements on the main diagonal are discarded since they only show the correlation of each element with itself which is always one. An example of desired elements of the correlation matrix, resulting correlation array and two possible orders of storing correlation values is shown in Figure 1.

In [19], a GPU-based tool was developed by Liang et al. for constructing gene co-expression networks based on computing N(N−1)/2 Pearson’s correlation coefficient. Wang et al. [20] proposed a hybrid CPU–GPU framework for computing Pearson’s correlations based on general matrix multiplication (GEMM). Their approach is based on the fact that Pearson’s correlation computation among two voxels can be reduced to vector dot product of their time series if each time series is normalized based on the following Equation:

(3) $$u_{i}={\frac{v_{i}-{\bar{v}}_{i}}{∥v_{i}-{\bar{v}}_{i}∥}}_{2}$$

In this Equation, $v_{i}$ is time series of voxel *i* and $u_{i}$ is the normalized time series of $v_{i}$. All normalized voxels are then aggregated in matrix $U(u_{1},u_{2},\ldots ,u_{N})$. Multiplying the matrix *U* to its transpose ($U\times U^{T}$) results the correlation matrix. Sometimes the size of correlation matrix is larger than GPU memory, in this case, their approach divides matrix U to smaller blocks and computes the multiplication of each block to others to cover all elements in upper triangle. After performing matrix multiplication of all blocks a post processing step is needed to reorder the elements and eliminate redundant correlations. This post-processing runs on CPU.

In [21] we proposed two GPU-based approaches to compute N(N−1)/2 Pearson’s correlation based on the order shown in Figure 1a. In the first approach, after normalizing the data using Equation (3), correlations of each voxel with the rest of voxels are computed by multiplying its time series to a matrix containing the time series of all other voxels. This process which is multiplying a vector to a matrix is continued for all voxels. The matrix that is multiplied to the time series of first voxel contains N−1 rows, for the second voxel, the matrix contains N−2 rows since correlation of first and second voxels have been computed by first matrix vector multiplication. Reducing the size of the matrix by one for each voxel, by performing N−1 matrix vector multiplication, the upper triangle part of correlation matrix (Figure 1a) is computed. The second approach, also introduced in [21], is called GPU-PCC and is based on performing vector dot product of normalized time series. In this technique, each 16 consecutive GPU threads are considered as a group and are responsible for performing vector dot product of two normalized time series which results in computing correlation between two voxels. In order to compute correlation coefficients in desired order, threads inside each group use the following mapping equations based on index of the group (*k*) to compute the index of two voxels (*i* and *j*) that their correlation should be stored at location *k*. Using these equations ensures that correlations are computed in order.

(4) $$i=n-2-\lfloor \frac{\sqrt{-8\times k+4\times n\times (n-1)-7}}{2}-0.5\rfloor$$

(5) $$j=k+i+1-\frac{n\times (n-1)}{2}+\frac{\left(n-i)\times ((n-i)-1\right)}{2}$$

If total size of correlation matrix is larger than GPU memory, this approach computes correlations until there is no free space in GPU, transfers the results to CPU and starts computing the rest of correlations. Our experiments on synthetic and real fMRI data showed that this approach can compute correlations faster than the first approach.

In this paper, we propose a GPU-based technique called Fast-GPU-PCC which computes correlation coefficients and reorders them. Both correlation computation and reordering steps are performed on GPU. We have introduced an out-of-core technique in order to make the GPU-based algorithm accessible for large data sets with an available architecture limited in memory. For our computations we performed the multiplication in multiple steps, where in each step, we multiply time series of a block of voxels to the remaining voxels. The post processing step is performed after matrix multiplication and then re-ordered computed correlations are stored in a correlation array. Our experiments show that we are able to perform correlations calculations up to 62 times faster than the CPU-version of the algorithms and up to 3 times faster than the GPU-based techniques. We discuss our proposed techniques in the sections below.

### GPU Architecture, CUDA Programming Model and cuBLAS Library

Processing a huge amount of data generated by fast and high-throughput instruments in the area of bioinformatics, biomedical and health care informatics is almost impossible using traditional and sequential CPU-based techniques. Many algorithms based on parallel computing techniques have been proposed in different fields like genomics, proteogenomics, clinical informatics, imaging informatics, etc. [22,23,24,25,26,27,28,29,30,31,32]. Using GPUs for accelerating these type of problems has become very popular recently. The very first goal of GPUs was satisfying demands for higher quality graphics in video games and creating more relalistic three-dimensional (3D) environment [33]. Nowadays, a multitude of high-performance applications exploit high throughput of enormous number of GPU cores [32,34]. A GPU consists of an array of streaming multiprocessors (SMs) each having multiple streaming processors or cores. New GPU devices house thousands of cores; e.g., NVIDIA Tesla K40 contains 2880 cores. On each core, hundreds of threads run based on the single instruction multiple thread (SIMT) strategy. Compute unified device architecture or CUDA is NVIDIA’s programming model interface designed for programming graphic cards to exploit parallelism. The function that is executed by GPU threads on a GPU device in parallel is called *kernel* function. The GPU threads are logically grouped into a programming abstraction called thread blocks and reside on the same processor core. The maximum number of threads per block is 1024 based on limited memory resources of the cores. Multiple blocks can be organized in one or two dimensions to form a grid. A collection of 32 consecutive threads is called a warp which is the smallest executable unit on GPU device. All threads in a warp perform the same instruction in a lock-step, concurrently. The GPU contains different memory types such as *global memory*, *shared memory*, *local memory*, and *registers*. Global memory is the main memory of GPU which is accessible by all threads. Data transferred from CPU to GPU resides on global memory. Shared memory on the other hand is on-chip memory which is shared among all threads within the same block, and is not accessible by threads in other blocks. Accessing data from shared memory is much faster than global memory and is efficient in case threads need to access data more than once. One important concept in designing GPU-based algorithms that should be taken into account is transferring data between CPU and GPU. Data transfer between CPU and GPU is a time consuming task based on the low bandwidth between CPU memory and GPU memory and some overhead that is associated to each data transfer. It is beneficial to minimize the number of transfers by combining small data transfers into one larger transfer when possible. Optimizing memory access is a useful strategy to exploit parallelism on GPU’s. Loading and storing data on device global memory can be coalesced into fewest possible transactions if all threads within the same warp access nearby locations of the memory, otherwise multiple transactions will be performed instead which deteriorates the efficiency. Thus, it is important to make sure threads inside a warp access data within the same locality. We tried to consider these concepts in designing our algorithm.

NVIDIA has provided efficient CUDA libraries such as CUDA basic linear algebra subroutines (cuBLAS) which performs vector and matrix operations like matrix multiplication and matrix vector multiplication [35]. In this study we used a built in function from this library which is very efficient for performing matrix multiplication.

## 2. Materials and Methods

As stated in the previous section, multiplying two vectors which are normalized by Equation (3) results in Pearson’s correlation between them. Normalizing all time series vectors takes much less time compared to multiplying pairwise time series, so we leave the normalization part to be performed on CPU. For the rest of the paper, let us assume that the time series of all voxels are stored in an N×M matrix called *U*, which *N* corresponds to the number of voxels and *M* corresponds to the number of data points of each voxel (length of time series).

After the data is normalized on the CPU, it is transferred to GPU global memory. Since the number of voxels are much more than the length of time series of each voxels, size of correlation matrix is very large and sometimes cannot be fitted inside GPU memory. In this case, correlation computation must be performed in multiple rounds such that in each round part of correlation coefficients should be calculated and transferred to CPU memory to free GPU space for the rest of computation. Additionally, our approach needs some extra space in GPU for storing reordered coefficients before transferring them back to CPU. If the total space that our algorithm needs is smaller than whole GPU memory, the algorithm can be run in one round, otherwise, multiple rounds are needed for completing the computations. In the next following sections, we first explain how to compute the space we need for computing correlation coefficients and reordering them inside GPU, then we go through two possible cases in which pairwise correlations can be computed in one round or several rounds.

### 2.1. Space Storage Needed for Computing Correlations and Reordering Them

**Figure 2:**
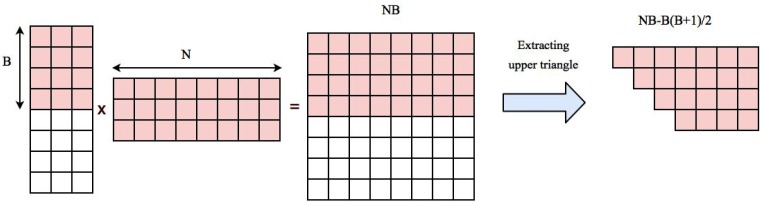
Space needed for computing correlation of first *B* voxels with the rest of voxels. Pairwise correlation is computed by multiplying a matrix containing time series of *B* voxels to a matrix containing time series of all voxels which results in a matrix containing N×B elements. This matrix has NB−B(B+1)/2 distinct correlation coefficients that need to be extracted and stored in the resulting correlation array.

Our approach is based on performing matrix multiplication and extracting the upper triangle part. Multiplying matrix U (N×M) to its transpose (M×N) generates $N^{2}$ Coefficients. Upper triangle part of the correlation matrix can be stored in an array with length $\frac{N(N-1)}{2}$. Normalized time series of voxels are transfered to GPU memory in the beginning of the algorithm and will stay there during the whole process. This will take an additional N×M space. So the total space needed for storing data, computing the correlation matrix and reordered correlation array in GPU is $N^{2}+\frac{N(N-1)}{2}+NM$. If this value is smaller than GPU memory, the whole computation can be done in one round, otherwise we first compute correlation of a block of data with *B* voxels to all other voxels, reorder and transfer them back to CPU and start a new block. The space needed for multiplying time series of *B* voxels to the rest of voxels is NB and extracted correlations belonging to upper triangle part of the correlation matrix corresponding to *B* blocks needs $NB-\frac{B(B+1)}{2}$. Figure 2 shows an example of these elements.

The total space needed for the computation is equal to $NM+NB+NB-\frac{B(B+1)}{2}$. The value of *B* should be chosen in such a way that the space needed for our computation is less than the free space in GPU memory at the time. Let us assume normalized time series of all voxels are already stored in GPU memory and the free space left is equal to *X*. Since the value of $NB-\frac{B(B+1)}{2}$ is smaller than *NB*, the upper bound of space we need is 2NB and value of *B* can be computed using the following equation

(6) $$B=\frac{X}{2N}$$

We compute the value of *B* in the beginning of our algorithm. If this value is greater than *N*, it means that the computation can be done in one round otherwise several rounds are needed for computation. In the next two subsections, we go through each case in detail.

### 2.2. Case 1: Correlation Computation Can Be Done in One Round

If the GPU has enough memory to store the whole correlation matrix and ordered correlation array, by multiplying matrix *U* to its transpose the whole correlation matrix is computed at once and we can extract the upper triangle part of the matrix. The idea that we used for extracting the upper triangle part is to assign one GPU thread to each cell of correlation matrix, if the cell is located in upper triangle above the diagonal, thread will copy its value to specific location in the correlation array. The index of each thread can be computed based on its thread ID, block ID and dimension of the block as follows:

(7) idx=blockDim.x∗blockIdx.x+threadIdx.x

After computing this index which is unique for each thread, we compute the row and column index of the cell that the thread is assigned to it. The row index and column index of each cell can be computed as quotient and remainder of dividing *idx* by *N*, i=idx/N and j=idx%N, respectively. *i* and *j* are indices of voxels which their correlation is stored at index (*i*,*j*) of the correlation matrix. In order to take the elements in upper triangle part of the matrix, elements with i<j are selected. Threads which are pointing to upper triangle part of the correlation matrix will save their corresponding correlations at index *k* of resulting correlation array which can be calculated as follows:

(8) $$k=i\times N-\frac{i\times (i+1)}{2}+j-i$$

**Figure 3:**
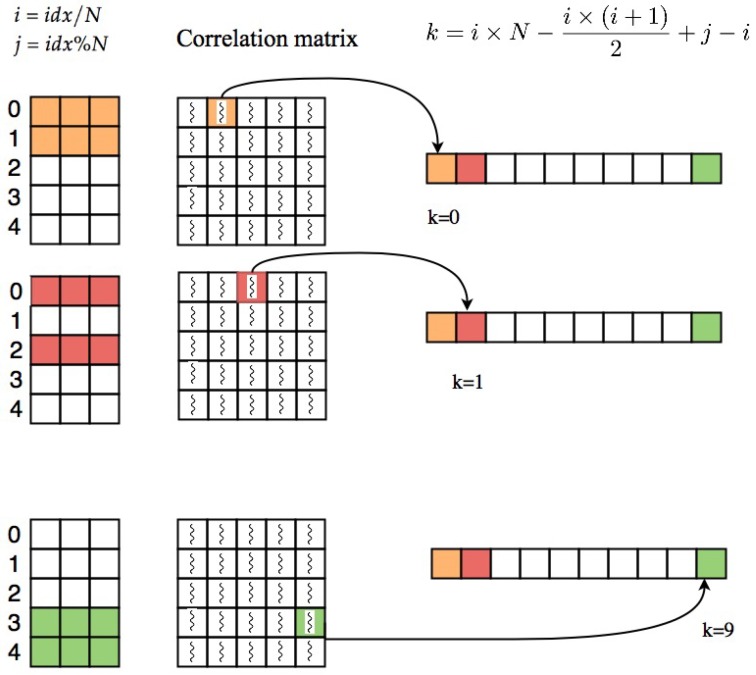
Process of extracting upper triangle part of correlation matrix based on Algorithm 1.

Using this equation, the coefficient will be saved in the correlation array based on the pattern shown in part a of Figure 1 (row major order). Figure 3 shows an example of this process.

The psudocode of reordering kernel is shown in Algorithm 1.

**Algorithm 1** Extracting ordered upper triangle part of correlation matrix**Input:**N×N correlation matrix *S***Output:** Ordered correlation array *C* of size N(N−1)/2 1: idx=blockDim.x∗blockIdx.x+threadIdx.x 2: i=idx/N 3: j=idx%N 4: **if**
i<j and i<N and j<N
**then** 5:  $k_{1}=i\times N-\frac{i\times (i+1)}{2}+j-i$ 6:  $k_{2}=j\times N+i$ 7:  $C\left[k_{1}-1\right]=S\left[k_{2}\right]$ 8: **end if**

After computing correlations and storing distinct pairs in correlation array, it will be copied to CPU memory.

### 2.3. Case 2: Correlation Computation Needs to Performed in Multiple Rounds

In cases that both correlation matrix and resulting array cannot be fitted inside GPU memory, the correlation of the first *B* voxels (*B* is computed using Equation (6)) to the rest of voxels are computed and reordered using Algorithm 2.

The reordering strategy is similar to Algorithm 1 with few changes since time series of a block of voxels (*B*) is multiplied to other voxels (*N*′). After reordering coefficients, results are transferred back to the CPU. A new block number should be calculated for computing the rest of coefficients. Since the correlation of the first *B* voxels with the rest of voxels are computed, new block number can be calculated using Equation (6) but this time using N−B instead of *N* in denominator. By doing this process, all correlation coefficients can be computed in multiple rounds.

**Algorithm 2** Extracting ordered upper triangle part of correlation matrix - case2**Input:**$B\times N^{\prime }$ correlation matrix *S***Output:** Ordered correlation array *C* of size $N^{\prime }B-B(B+1)/2$ 1: idx=blockDim.x∗blockIdx.x+threadIdx.x 2: $i=idx/N^{\prime }$ 3: $j=idx\%N^{\prime }$ 4: **if**
i<j and i<B and $j<N^{\prime }$
**then** 5:  $k_{1}=i\times N^{\prime }-\frac{i\times (i+1)}{2}+j-i$ 6:  $k_{2}=j\times B+i$ 7:  $C\left[k_{1}-1\right]=S\left[k_{2}\right]$ 8: **end if**

### 2.4. Overall Algorithm

Considering both cases, Algorithm 3 shows the overall scheme of our proposed method.

**Algorithm 3** Fast-GPU-PCC**Input:**N×M matrix *U* of time series data**Output:** Correlation array *C* of size N(N−1)/21:Preprocess the fMRI data using Equation (3)2:Copy normalized data to GPU global memory3:
B=X/2N
4:
**if**
B>N
**then**
5:  Multiply matrix *U* to its transpose $U^{T}$6:  Extract upper triangle part of the matrix using Algorithm 17:  Transfer the correlation array to CPU8:
**else**
9:  i=0, $N^{\prime }=N$10:  **while**
i<N
**do**11:    Multiply rows *i* to i+B of matrix *U* to columns *i* to *N* of $U^{T}$12:    Extract the upper triangle part of correlation matrix using Algorithm 213:    Transfer the extracted correlations to CPU14:    i=i+B15:    $N^{\prime }=N^{\prime }-B$16:    $B=X/2N^{\prime }$17:    **if**
$B>N^{\prime }$
**then**18:      $B=N^{\prime }$19:    **end if**20:  **end while**21:
**end if**


**Figure 4:**
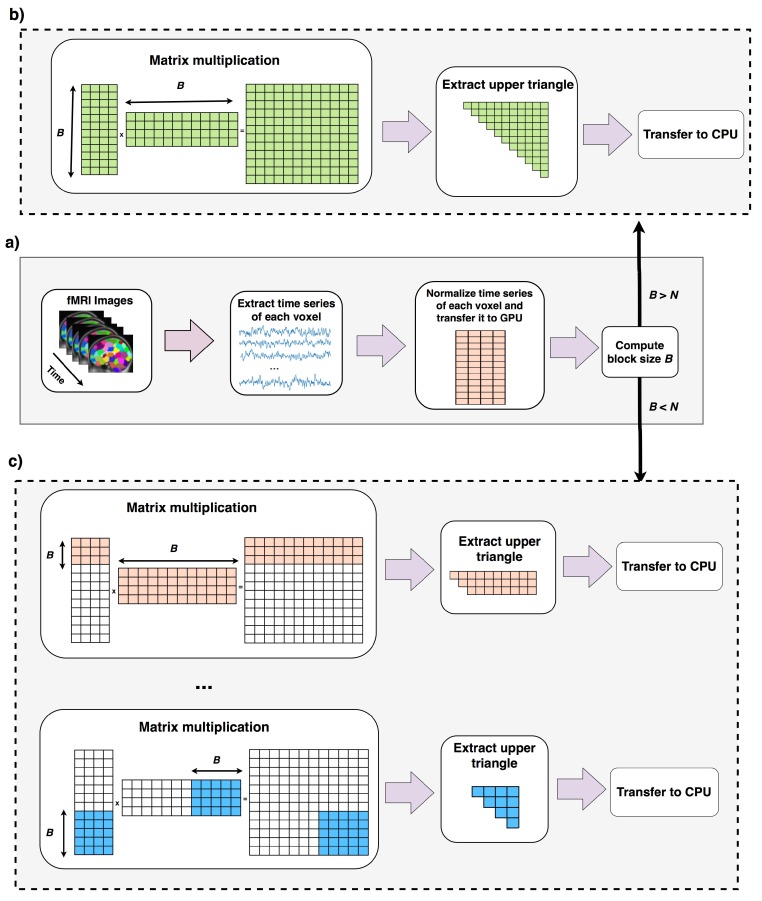
Overall process of Fast-GPU-PCC. In part (**a**), fMRI time series is normalized in CPU and transferred to GPU memory. Block size *B* is computed using Equation (6). If *B* is larger than *N* means that the whole computation can be performed in one round which is shown in part (**b**). In part (**b**) the whole normalized matrix is multiplied to its transpose and upper triangle is extracted and transferred back to CPU. If block size is computed in part (**a**) is smaller than *N* means that only pairwise correlation of *B* voxels with the rest of voxels can be computed which is shown in part (**c**). In part (**c**) after correlation of the first *B* voxels with the rest of voxels is computed and transferred back to CPU, new block size is computed and this process is repeated multiple time until all pairwise correlations are computed.

Data is preprocessed and copied to GPU memory (lines 1, 2). Lines 4–7 run when the total computation can be done in one round as explained in Section 2.2. Lines 9–20 runs when computation cannot be done in one round (Section 2.3). In this case correlation of *B* voxels (*B* is computed in line 3) to the rest of voxels are computed, reordered and copied back to CPU. In line 16, new size of B is computed using equation 6 this time ignoring the first *B* voxels. A new variable called *N*′ stores the number of remaining voxels that their pairwise correlations to the rest of voxels should be computed. If block size *B* is greater than *N*′, shows the case that pairwise correlation of the rest of elements can be done in one round, otherwise this process should be continued for more rounds. The overall process of this algorithm is shown in in Figure 4.

## 3. Results

All the experiments of this section are performed on a Linux server with Ubuntu Operating System version 14.04. This server includes two Intel Xeon E5 2620 processors with clock speed 2.4 GHz, 48 GB RAM and NVIDIA Tesla K40c Graphic Processing Unit. The GPU contains 15 streaming multiprocessors each consists of 192 CUDA cores and 11,520 MB global memory. The implemented code is available as General Public License (GPL) license on GitHub portal of our lab [36]. We have compared our method with three other methods. The first method is sequential version of computing pairwise PCC. The second method is GPU-PCC [21] algorithm which is a GPU-based technique able to compute Pearson’s correlations in order and the third method is proposed by Wang et al. [20]. In Wang’s method they compute pairwise correlations by performing matrix multiplications on GPU multiple times and in order to reorder the correlation coefficients and eliminate redundant ones, the results is post processed on CPU. We considered the time of both matrix multiplication and post-processing steps. All the experiments for each dataset are repeated multiple times and the minimum running time for each approach is reported. Optimization level -O2 was used for compiling codes that run on CPU. CUDA version 7.5 and g++ version 4.8.4 are used for our experiments. For all approaches we start measuring time right before preprocessing started until all correlations are computed and resided in CPU. We compared the scalability of our method with other methods by increasing the number of voxels and increasing the length of time series. The following sections explain the experiments in more detail.

### 3.1. Increasing Number of Voxels

**Figure 5:**
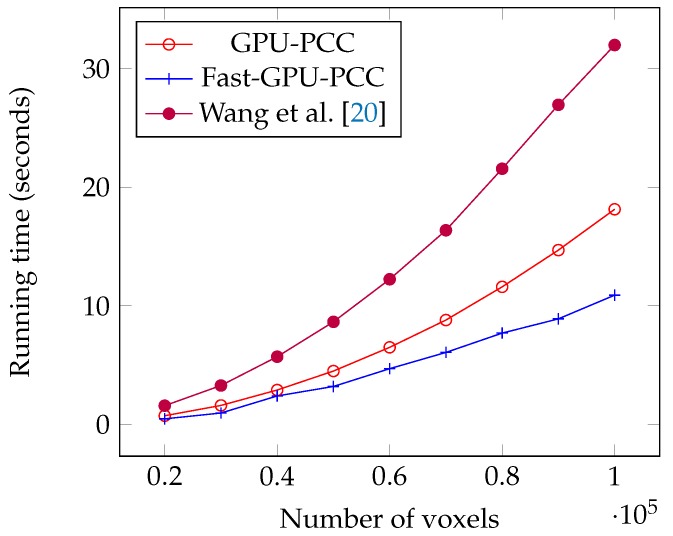
Running time comparison of Fast-GPU-PCC with other GPU-based techniques.

**Figure 6:**
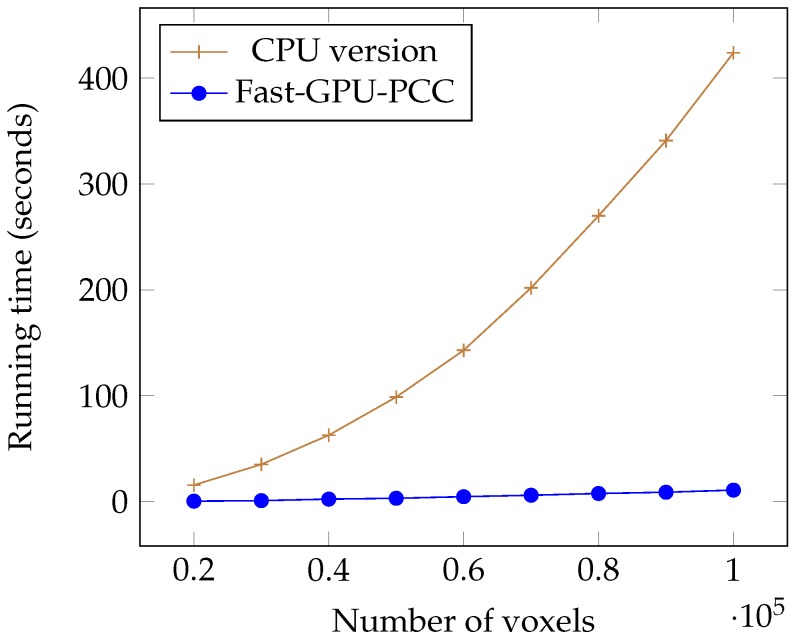
Running time comparison of Fast-GPU-PCC and CPU version.

Today, fMRI scanners are able to provide high resolution images in which we are dealing a huge amount of voxels. To ensure our method is able to handle large number of voxels we performed an experiment considering different number of voxels from 20,000 to 100,000 each having a time series of length 100. We used a synthetic dataset for this experiment. For each voxel, we generated a vector of 100 uniformly random floating point numbers in the range −6 and 6 as the intensity of each voxel. Table 1 shows the running time of each method based on different number of voxels in seconds. We also plotted the running times of all GPU-based techniques in Figure 5 and compared the running time of Fast-GPU-PCC and sequential version in Figure 6 (we used a different figure for this comparison since having sequential version with other techniques in the same figure makes comparison of GPU-based techniques difficult). As we see in Figure 5 and Figure 6, Fast-GPU-PCC runs faster than other techniques for all values of *N*. Speedup of Fast-GPU-PCC compared to other techniques are shown in Table 2. The speed up over CPU version, GPU-PCC and Wang’s technique is about 30, 1.5 and 3 times, respectively.

### 3.2. Increasing the Length of Time Series

**Figure 7:**
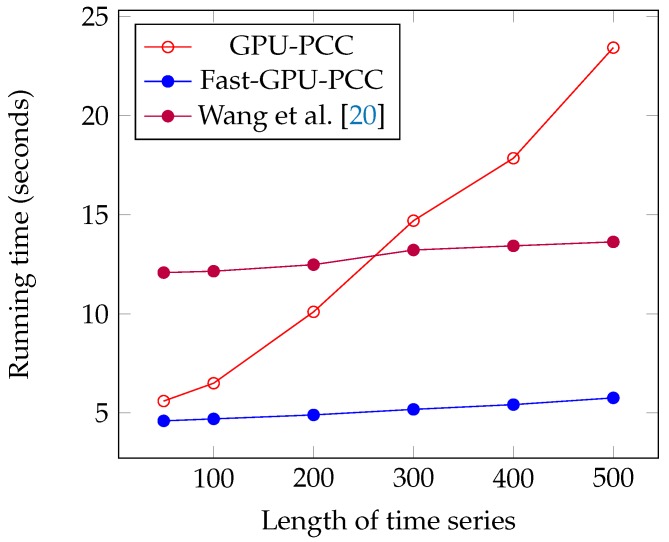
Running time comparison of Fast-GPU-PCC with other GPU-based techniques.

**Figure 8:**
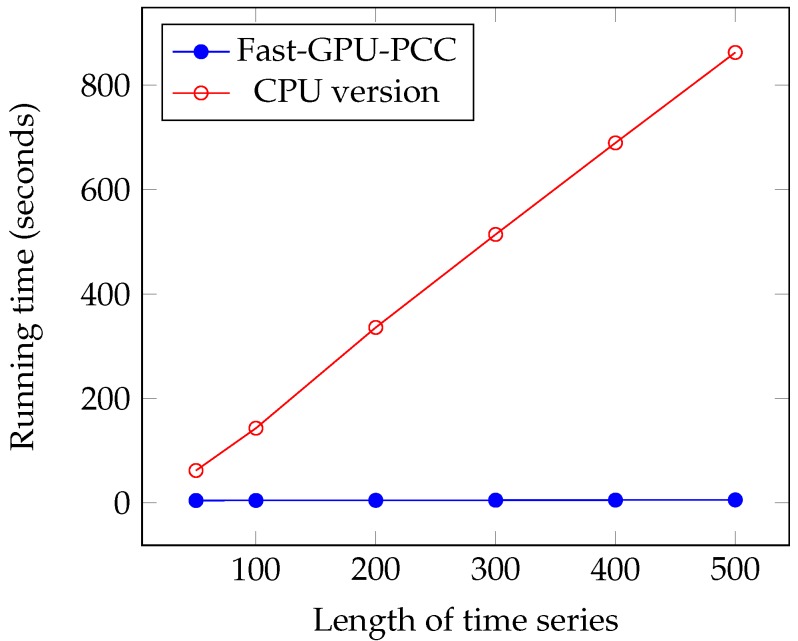
Running time comparison of Fast-GPU-PCC and CPU version.

We performed another experiment to measure the running time of our approach by increasing the length of the time series. The data that we used in this section is also synthetic data. To observe how increasing the length of time series affect the running time, we performed our experiment by considering fixed 60,000 voxels and each time changed the length of time series. We measured the running time for 50, 100, 200, 300, 400 and 500 time points in each time series. Similar to the previous section, a uniformly random floating point number in the range −6 and 6 is used as intensity of each voxel. Figure 7 and Figure 8 and Table 3 show the running time of different approaches in seconds and the speed ups gained by Fast-GPU-PCC compared to other approaches is shown in Table 4.

Like increasing the number of voxels, Fast-GPU-PCC runs faster than other techniques by increasing the length of time series. Speed up over Wang’s technique is about 2.5 times faster for all values of *M*. Speed up over GPU-PCC and CPU version increases as we increase the length of time series. It starts from 1.21× and 13.47× for M=50 and reaches to 4.04× and 149.72× for M=500. By increasing the length of time series, a slight increase in running time for Fast-GPU-PCC and Wang’s technique is observed. Both these approaches use the cublasSgemm() function from cuBLAS library for performing matrix multiplication which has highly optimized implementation. Therefore, increasing the length of time series while keeping the number of voxels constant does not increase the multiplication time significantly. Furthermore, due to the constant number of voxels, the size of the correlation matrix does not change, so the time spent on our reordering kernel and transferring correlations to CPU remains constant. Since most of the time is being spent on computing correlations, reordering and transferring them back to CPU increasing the length of time series does not affect the total running time.

### 3.3. Experiments on Real Experimental Data

We performed another experiment on real fMRI data and measured the running time of all techniques. The dataset we used is called Orangeburg dataset [37] which is part of 1000 Functional Connectomes Project public dataset. All subjects in this dataset are anonymous and no protected health information is included. It consists of resting state fMRI data of 20 healthy subjects, 5 male and 15 female with age range 20–55. We picked a random subject from this dataset for our experiment. The number of voxels in this dataset is equal to 90,112 and length of time series is equal to 165. Table 5 and Table 6 show the running time comparison and speed up achieved by Fast-GPU-PCC over other methods.

Similar to synthetic data, FAST-GPU-PCC runs faster than other techniques on real data. It runs about 3 times faster than Wang’s technique, 2.24 times faster than GPU-PCC and 62.3 times faster than CPU version.

## 4. Discussion

Pearson’s correlation coefficient is a very well used technique in fMRI data analysis for studying functional connectivity of the brain. fMRI images contain thousands of voxels and using traditional techniques for computing pairwise Pearson’s correlation is very time consuming. Therefore, using parallel computing techniques is essential for processing data- and computational-intensive operations like the computing correlation for big brain research.

In this paper, we proposed a GPU-based technique called Fast-GPU-PCC which computes correlation coefficients and reorders them in two possible ways. Both correlation computation and reordering steps are performed on GPU. The size of the correlation matrices are usually larger than total space in GPU memories. Fast-GPU-PCC is able to perform the whole computation in multiple steps based on memory limits of GPU. In this case, our strategy performs multiplication in multiple steps, where in each step, we multiply time series of a block of voxels to the remaining voxels. The post processing step is performed right after each matrix multiplication after which the results are reordered, stored in the resulting correlation array and transferred to CPU.

We performed several experiments on synthetic and real fMRI data and compared it with two other state-of-the-art CPU- and GPU-based techniques. All of these approaches yield the same for correlation. During our experiments on synthetic data, we investigated the effects of increasing the number of voxels and length of time series on scalability of Fast-GPU-PCC. To see how scalable Fast-GPU-PCC is in terms of number of voxels, we began by using 20,000 voxels and continued our process to 100,000 voxels. Fast-GPU-PCC outperformed all other techniques for all sizes and achieved up to 3 times speed up compared to other GPU-based techniques and more than 30 times compared to CPU version. Based on this experiment, the correlation computation of cases with more than 40,000 voxels are performed in multiple rounds. This strategy assures that our algorithm can be used on GPU devices with less memory as compared to the data that needs processing. In another experiment, we investigated the effect of increasing length of time series from 50 to 500. Fast-GPU-PCC out-performed other techniques such that its speed up increased over CPU version and one of GPU-based techniques and ran about 2.5 times faster compared to another GPU-based technique. Experiments on real data containing about 90,000 voxels also showed a promising result for Fast-GPU-PCC such that it ran up to 3 times faster than other GPU-based techniques and ≈62 times faster than CPU version. Such a high-scalability of our proposed GPU-based technique is ideal of big fMRI experiments. The speedups for computing fMRI data can have a significant impact on neurological and clinical research endeavors, especially for studying the functional connectives of large number of subjects. Our parallel approach helps reducing the compute time needed for constructing functional associations of each brain which results in reducing the resources required for large systems wide studies. Furthermore, storing correlations in an ordered fashion makes them easy to access for future use without much pre-processing. A number of research problems remains open and the techniques introduced in this manuscript suggest new directions of research that can be pursued. For future direction of this study we will focus our attention on dynamic functional networks from fMRI studies. Studying the dynamic behavior of functional connectivity in brain has become popular in order to understand variations of connectivity among different voxels. Large dimensionality of fMRI data pushes the researchers to use regions of interest instead of all voxels which can negatively influence the final results due to loss of fine-grain information. Designing GPU-based algorithms can be very helpful in order to reduce the time of this process in the voxel-level analysis. The other challenge in this area would be the huge space that is required for storing correlation matrices since large matrices need much more memory than that which is available in mid-sized labs. To this end, we will focus on designing fast compression algorithms for reducing the size of correlation matrices in such a way that compressed values can be used for further analysis without decompression. Thus, we believe that Fast-GPU-PCC is a very useful GPU-based technique to compute Pairwise Pearson’s Correlation Coefficients for fMRI data and studying the functional connectivity of the brain. We believe that the proposed parallel algorithm will be immensely useful to the neuroscience, psychiatry, fMRI and parallel computing communities.

## Figures and Tables

**Table 1:** Comparing running time (seconds) of different approaches on synthetic fMRI data.

Number of Voxels (*N*)	GPU-PCC	Fast-GPU-PCC	Wang et al. [20]	CPU Version
20,000	0.73	0.47	1.58	15.65
30,000	1.6	0.96	3.28	35.23
40,000	2.9	2.40	5.71	62.81
50,000	4.5	3.2	8.65	98.8
60,000	6.5	4.7	12.15	143
70,000	8.8	6.07	16.2	202
80,000	11.6	7.7	21.56	270
90,000	14.7	8.9	26.95	341
100,000	18.14	10.9	31.99	424

**Table 2:** Speed up gained by Fast-GPU-PCC over other methods by increasing the number of voxels.

Number of Voxels (*N*)	GPU-PCC	Wang et al. [20]	CPU Version
20,000	1.55	3.36	33.29
30,000	1.66	3.41	36.69
40,000	1.2	2.37	26.17
50,000	1.4	2.7	30.8
60,000	1.38	2.58	30.42
70,000	1.4	2.66	33.27
80,000	1.5	2.8	35.06
90,000	1.65	3.02	38.31
100,000	1.66	2.93	38.89

**Table 3:** Running time comparison of Fast-GPU-PCC with and GPU-based techniques.

Length of Time Series (*M*)	GPU-PCC	Fast-GPU-PCC	Wang et al. [20]	CPU Version
50	5.6	4.6	12.08	62
100	6.5	4.7	12.15	143
200	10.08	4.9	12.48	335.9
300	14.7	5.18	13.22	514
400	17.85	5.42	13.43	689.3
500	23.32	5.76	13.63	862.4

**Table 4:** Speed up gained by Fast-GPU-PCC over other methods by increasing length of time series.

Length of Time Series (*M*)	GPU-PCC	Wang et al. [20]	CPU Version
50	1.21	2.62	13.47
100	1.38	2.58	30.4
200	2.05	2.54	68.5
300	2.83	2.55	99.22
400	3.29	2.47	127.12
500	4.04	2.36	149.72

**Table 5:** Running time comparison of Fast-GPU-PCC with other techniques on real fMRI data.

Fast-GPU-PCC	GPU-PCC	Wang et al. [20]	CPU Version
9.26	20.83	27.46	577

**Table 6:** Speed up gained by Fast-GPU-PCC over other methods on real fMRI data.

GPU-PCC	Wang et al. [20]	CPU Version
2.24	2.96	62.3
